# Supplementary material for: A gonad-expressed opsin mediates light-induced spawning in the jellyfish Clytia
Source: eLife. 2018 Jan 5;7:e29555. doi: 10.7554/eLife.29555 (PMC5756024; doi:10.7554/eLife.29555)
Supplement: Transparent reporting form [file elife-29555-transrepform.docx]

***eLife’s* transparent reporting form**

We encourage authors to provide detailed information *within their submission* to facilitate the interpretation and replication of experiments. Authors can upload supporting documentation to indicate the use of appropriate reporting guidelines for health-related research (see [EQUATOR Network](http://www.equator-network.org/%20)), life science research (see the [BioSharing Information Resource](https://biosharing.org/)), or the [ARRIVE guidelines](http://www.plosbiology.org/article/info:doi/10.1371/journal.pbio.1000412) for reporting work involving animal research. Where applicable, authors should refer to any relevant reporting standards documents in this form.

If you have any questions, please consult our Journal Policies and/or contact us: [editorial@elifesciences.org](mailto:editorial@elifesciences.org).

**Sample-size estimation**

- You should state whether an appropriate sample size was computed when the study was being designed
- You should state the statistical method of sample size computation and any required assumptions
- If no explicit power analysis was used, you should describe how you decided what sample (replicate) size (number) to use

Please outline where this information can be found within the submission (e.g., sections or figure legends), or explain why this information doesn’t apply to your submission:

For the comparison of gonad spawning responses between mutant and wild type medusa (illustrated in Fig. 3D/E) sample sizes were not computed, but were appropriate for, and greatly exceeded, the formal requirements for the statistical test performed (Fisher’s exact test – details in Figure 3 legend).

For the comparison of quantified MIH immunofluorescence in gonad neurons between mutant and wild type medusa, before and after light stimulation (illustrated in Fig. 4) sample sizes were not computed, but were appropriate and greatly exceeded the formal requirements for the statistical test performed (Man Whitney U test-see legend).

For determining the spectral response of *Clytia* ovaries (illustrated in Fig.1B), no statistical comparisons were made between wavelengths. The numbers of gonads examined and experimental repeats (see below) were sufficient to support the conclusions about peak activating wavelengths as presented.

**Replicates**

- You should report how often each experiment was performed
- You should include a definition of biological versus technical replication
- The data obtained should be provided and sufficient information should be provided to indicate the number of independent biological and/or technical replicates
- If you encountered any outliers, you should describe how these were handled
- Criteria for exclusion/inclusion of data should be clearly stated
- High-throughput sequence data should be uploaded before submission, with a private link for reviewers provided (these are available from both GEO and ArrayExpress)

Please outline where this information can be found within the submission (e.g., sections or figure legends), or explain why this information doesn’t apply to your submission:

For the spectral characterisation of the spawning response of isolated *Clytia* ovaries (illustrated in Fig.1B), a total of 5-6 experiments were performed (biological replicates), using 3-6 isolated gonads per condition and experiment (technical replicates). Outliers are depicted in Fig.1B as empty circles. These details are included in the Figure legend and in Methods “Monochromator assay”).

The biological replicates for RNA-seq samples (2 for each sample) consisted of pooled tissues and oocytes from gonads dissected on different days, under the same conditions and same jellyfish age (see Methods – gonad transcriptome analysis).

The transcriptome data have been deposited in NCBI's Gene Expression Omnibus and are accessible through GEO Series accession number GSE101072 .

https://www.ncbi.nlm.nih.gov/geo/query/acc.cgi?acc=GSE101072

Reviewer token: yzclkwgkztkptqf- NOTE Data will be released publicly one the manuscript is accepted for publication

*Clytia* opsin amino GenBank numbers are provided in the Methods section (Key resources table) .

Single *in situ* hybridization experiments were performed 3 times and double *in situ* hybridization experiments were performed twice. Quantification of Opsin9 - PP4 co-expression was performed by counting 594 cells from 10 different gonads obtained in a single experiment. All these details are described in Methods under “*in situ* hybridization”.

For the comparison of gonad spawning responses (illustrated in Fig. 3D/E), three independent experiments were performed (biological replicates), using 80 to 154 isolated gonads of wild type or *Opsin9*^n1_4^ strains in total (technical replicates). These details are included in the figure legend and in the Methods under “Gonad spawning assays”.

For the comparison of MIH immunofluorescence in gonad neurons (illustrated in Fig. 4), two independent experiments were performed (biological replicates) and clearly gave equivalent results visually. Quantification of fluorescence was performed one experiment. Between 183 and 282 cells were analyzed from 5-6 independent gonads (technical replicates) of wild type or *Opsin9*^n1_4^ strains under each experimental condition. All details are described in Methods under “Immunofluorescence ”.

All experimental data obtained are included in the manuscript except:

- Initial Opsin Phylogeny trials using different choices of evolutionary model and taxon sample (p7), and some AU phylogenetic test trials (p17).

- Probe cross-reaction controls for double In situ hybridization experiments.

**Statistical reporting**

- Statistical analysis methods should be described and justified
- Raw data should be presented in figures whenever informative to do so (typically when N per group is less than 10)
- For each experiment, you should identify the statistical tests used, exact values of N, definitions of center, methods of multiple test correction, and dispersion and precision measures (e.g., mean, median, SD, SEM, confidence intervals; and, for the major substantive results, a measure of effect size (e.g., Pearson's r, Cohen's d)
- Report exact p-values wherever possible alongside the summary statistics and 95% confidence intervals. These should be reported for all key questions and not only when the p-value is less than 0.05.

Please outline where this information can be found within the submission (e.g., sections or figure legends), or explain why this information doesn’t apply to your submission:

Statistical methods are described and justified in Methods under “Graphs and statistics”. Details of the statistical tests performed are provided in the legends to Figures 3 and 4.

The number of samples included in each experimental group (n) is stated in the legends to Figure 1, 3 and 4 legends, and was greater than 10 in all cases.

(For large datasets, or papers with a very large number of statistical tests, you may upload a single table file with tests, Ns, etc., with reference to sections in the manuscript.)

**Group allocation**

- Indicate how samples were allocated into experimental groups (in the case of clinical studies, please specify allocation to treatment method); if randomization was used, please also state if restricted randomization was applied
- Indicate if masking was used during group allocation, data collection and/or data analysis

Please outline where this information can be found within the submission (e.g., sections or figure legends), or explain why this information doesn’t apply to your submission:

Dissected gonads for each experiment were allocated to different groups randomly upon dissection.

**Additional data files (“source data”)**

- We encourage you to upload relevant additional data files, such as numerical data that are represented as a graph in a figure, or as a summary table
- Where provided, these should be in the most useful format, and they can be uploaded as “Source data” files linked to a main figure or table
- Include model definition files including the full list of parameters used
- Include code used for data analysis (e.g., R, MatLab)
- Avoid stating that data files are “available upon request”

Please indicate the figures or tables for which source data files have been provided:

Figure 2 source data 1 provides numerical data (normalized counts) used to construct the heat map shown in Figure 2A.

Sequences of guide RNAs and genotyping primers used for generating *Opsin9* mutant *Clytia* are provided in Figure3 source data .

Sequence alignments for Figure 6 and Figure 6 supplement 1 and 2 are provided in the four Figure 6 source files
